# Assessment of Postoperative Complications Between Interrupted Modified Smead-Jones Rectus Closure and Conventional Rectus Closure in Midline Laparotomy Wounds: A Comparative Study

**DOI:** 10.7759/cureus.72712

**Published:** 2024-10-30

**Authors:** Aiswerya Shankar, B. V. Sreedevi, Sundeep Selvamuthukumaran, Sunidhi Rajput, Pola Govardhan Kumar

**Affiliations:** 1 General Surgery, Sree Balaji Medical College & Hospital, Chennai, IND; 2 General Surgery, Sri Ramaswamy Memorial (SRM) Medical College Hospital and Research Centre, Kanchipuram, IND

**Keywords:** far-near-near-far suture, midline laparotomy, midline laparotomy incision, rectus closure, burst abdomen

## Abstract

Introduction

The abdomen comprises several layers that enhance its strength and flexibility, extending from the superficial to the deep layers of the anterior abdominal wall. Among these, the rectus sheath serves as a fibromuscular compartment essential for structural integrity, providing mechanical support and safeguarding the underlying tissues and organs. This study evaluates two midline closure techniques employed after midline laparotomy surgery to determine which technique offers superior strength and mechanical support.

Materials and methods

A single-center prospective comparative study was conducted over 18 months, from October 2022 to April 2024, at Sree Balaji Medical College & Hospital in Chennai, India. The study population comprised 92 outpatients and inpatients from the general surgery and emergency departments who underwent emergency and elective midline laparotomy surgery. Patients were selected using simple random sampling and categorized into two groups based on the type of rectus closure: conventional rectus closure (CRC) and interrupted modified Smead-Jones (IMSJ) rectus closure.

Results

In our study, approximately 34% (N = 31) of participants were aged 46-60 years, with a mean age of 53.45 ± 7.98 years. The sex distribution did not show significant preponderance. Complications included three cases of burst abdomen postoperatively, with 66.67% (N = 2) occurring in the CRC group. Incisional hernias were observed in six cases, with five belonging to the CRC group, indicating statistical significance. Additionally, wound infections were recorded in 10 cases, all within the CRC group. Out of 20 patients who developed localized collections, 32.6% (N = 15) were in the CRC group, while five belonged to the IMSJ group.

Conclusions

This study demonstrates that the IMSJ technique is superior to CRC in reducing postoperative complications. This advantage is attributed to its enhanced tensile strength and improved distribution of tension across the wound.

## Introduction

Midline laparotomy is a commonly employed surgical technique for abdominal procedures, continuously reviewed and refined to improve patient outcomes and minimize postoperative complications, such as wound dehiscence and hernia formation. The effectiveness and safety of surgical techniques in reducing these complications are critical in surgical practice. This study investigates two midline closure techniques: the Interrupted modified Smead-Jones (IMSJ) rectus closure and the conventional rectus closure (CRC), focusing on their impacts on surgical outcomes, including complications.

The IMSJ closure technique incorporates the full thickness of the anterior abdominal wall using interrupted sutures, aiming to provide greater tensile strength and reduce stress on the incision. This approach theoretically decreases the likelihood of wound dehiscence and hernia development, potentially leading to improved patient outcomes [1]. Conversely, the CRC technique typically employs a continuous suture method, with variations in suture depth and distance from the wound, and has been a traditional choice for many surgeons [2]. The ongoing debate surrounding interrupted versus continuous suturing techniques for abdominal closures involves considerations such as wound healing, infection rates, and closure strength [3]. This study aims to comprehensively evaluate and present empirical evidence regarding postoperative complications, including localized fluid collection, wound dehiscence, and hernia development, associated with these two closure techniques following midline laparotomy.

## Materials and methods

Study design

This prospective comparative study included outpatients and inpatients from the Department of General Surgery and the Emergency Department at Sree Balaji Medical College & Hospital in Chennai, India. A simple random sampling method was employed, whereby each patient was assigned to one of the two groups based on randomly generated computer numbers, ensuring equal chances of allocation to either group. With a confidence interval of 95%, the sample size was determined to be 92, comprising 46 participants in each group. Study Group 1 underwent the CRC technique for closing rectus incisions, while Group 2 received the IMSJ technique. The aim of the study was to assess and compare postoperative complications between the two groups and to analyze the association of demographic variables with postoperative complications.

Ethical considerations

Patients were informed about the study, and informed consent was obtained from all participants prior to their inclusion. Institutional approval was granted by the Institutional Human Ethical Committee at the Department at Sree Balaji Medical College & Hospital during a meeting held on September 22, 2022 (approval number 002/SBMCH/IHEC/2022/1855).

Study criteria

The inclusion criteria for the study comprised individuals aged 18 years and older who had midline incisions from emergency or elective laparotomy. Exclusion criteria included patients with a survival prognosis of less than one week due to complications, those who underwent surgeries other than midline incisions, and patients requiring re-laparotomy.

Methods

Based on the inclusion and exclusion criteria, patients were assigned to two groups to undergo either the IMSJ closure or the CRC. After surgery, patients were monitored as inpatients for the first week for complications, including localized fluid collection, wound infections, and burst abdomen. Following discharge from the hospital, the study groups were followed up at six weeks and six months to assess for incisional hernia.

Measurement of outcome variables

The effectiveness of both techniques was assessed during the postoperative phase based on the frequency of localized fluid collection, wound infection, burst abdomen, and incisional hernia.

Operative definition

CRC involves the continuous closure of the rectus fascia, with suture bites taken 10 mm from the edge of the fascia using 1-polypropylene sutures. In contrast, the IMSJ technique entails closing the rectus fascia in an interrupted manner, utilizing a far-near-near-far pattern. In this technique, the entry and exit bites are approximately 15-20 mm away from the suture line, with the sutures placed about 10 mm from the edge of the rectus fascia, also using 1-polypropylene sutures.

Statistical analysis

A detailed history was collected from all patients, and clinical and demographic data were compiled. The data were entered into a Microsoft Excel spreadsheet (Microsoft Corporation, Redmond, WA, USA), and statistical analysis was conducted using IBM SPSS Statistics for Windows, Version 22.0 (Released 2013; IBM Corp., Armonk, NY, USA). Frequencies and proportions were calculated for categorical data, while means and standard deviations were reported for continuous variables. A p-value of less than 0.05 was considered statistically significant.

## Results

The study was conducted with 92 patients to compare the effectiveness of CRC and IMSJ techniques in midline closure based on the incidence of localized fluid collection, wound infection, burst abdomen, and incisional hernias in the postoperative period. Patients were randomly assigned to two groups: Group 1 underwent CRC, while Group 2 received IMSJ.

Table 1 presents the age distribution of the total population, highlighting the highest number of cases (N = 31) in the 46-60 years age group, followed by 30 cases in the 31-45 years age group. Table 2 illustrates the cross-tabulation of age across the two groups. The p-value of 0.003 indicates a statistically significant association between age and group membership. Specifically, younger individuals (up to 30 years) were evenly distributed between the two groups. However, the proportion of patients in Group 2 increased in the 31- to 45-year age range, with 76.7% belonging to this group. In contrast, the trend reversed for patients aged 46-60 years and those above 60, with a larger proportion belonging to Group 1 (67.7% and 63.2%, respectively).

**Table 1:** Distribution of age in the total study population

Age	Number of cases (N)	Percentage (%)
Up to 30 years	12	13
31-45 years	30	33
46-60 years	31	34
Above 60 years	19	21
Total	92	100
Mean ± SD	53.45 ± 7. 98 years	

**Table 2:** Comparison of age between Group 1 and Group 2 Inferential statistical analysis using Pearson’s chi-square test p < 0.05 is statistically significant.

Age * Group cross-tabulation						Chi-square value	p-value
			Group		Total		
			Group 1	Group 2		13.752	0.003
Age	Up to 30 years	Count (N)	6	6	12		
		% within age	50%	50%	100%		
	31-45 years	Count (N)	7	23	30		
		% within age	23.3%	76.7%	100%		
	46-60 years	Count (N)	21	10	31		
		% within age	67.7%	32.3%	100%		
	Above 60 years	Count (N)	12	7	19		
		% within age	63.2%	36.8%	100%		
Total		Count (N)	46	46	92		
		% within age	50%	50%	100%		

Table 3 presents the distribution of cases by sex across the two groups. In Group 1, there are 14 males, while Group 2 has 18 males, representing 43.8% and 56.3% of the male population, respectively. For females, 32 (53.3%) are in Group 1, and 28 (46.7%) are in Group 2. The p-value of 0.381 indicates that the difference in sex distribution between the groups is not statistically significant.

**Table 3:** Comparison of sex between Group 1 and Group 2 Inferential statistical analysis using Pearson’s chi-square test p <0.05 is statistically significant

Sex * Group cross-tabulation						Chi-square value	p-value
			Group		Total		
			Group 1	Group 2		0.767	0.381
Sex	Male	Count (N)	14	18	32		
		% within sex	43.80%	56.30%	100%		
	Female	Count (N)	32	28	60		
		% within sex	53.30%	46.70%	100%		
Total		Count (N)	46	46	92		
		% within sex	50.00%	50.00%	100%		

**Figure 1:**
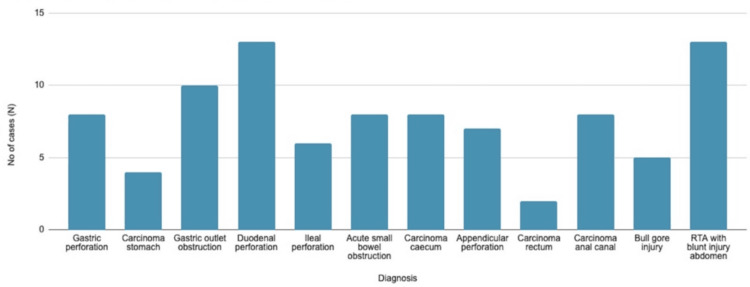
Distribution of diagnosis across the study population

Table 4 and Figure 1 illustrate the distribution of diagnoses within the study population. The conditions with the highest number of cases include duodenal perforation and road traffic accidents (RTAs) resulting in blunt abdominal injuries, each accounting for 13 cases. The rarest condition reported is rectal carcinoma with large bowel obstruction, with only two cases. Overall, the data reveals a diverse range of diagnoses related to abdominal injuries, with perforation of a hollow viscus organ being the most prevalent, totaling 34 cases.

**Table 4:** Relative diagnosis distribution in the study population RTA, road traffic accident

Diagnosis	Number of cases (N)	Percentage (%)
Gastric perforation	8	8.7
Carcinoma stomach	4	4.35
Gastric outlet obstruction due to cicatrized duodenal ulcer	10	10.87
Duodenal perforation	13	14.13
Ileal perforation	6	6.52
Acute small bowel obstruction	8	8.7
Carcinoma caecum	8	8.7
Appendicular perforation	7	7.61
Carcinoma rectum with large bowel obstruction	2	2.17
Carcinoma anal canal with large bowel obstruction	8	8.7
Bull gore injury	5	5.43
RTA with blunt injury to the abdomen	13	14.13
Total	92	100

Table 5 details the cross-tabulation of comorbidities between the two groups. Out of 24 individuals with comorbidities, 11 are in Group 1 and 13 in Group 2, translating to 45.8% and 54.2%, respectively, within these categories. The p-value of 0.635 suggests that the presence or absence of comorbidities does not significantly affect the distribution between the two groups.

**Table 5:** Comparison of comorbidities between Group 1 and Group 2 Inferential statistical analysis using Pearson’s chi-square test p < 0.05 is statistically significant

Comorbidities * Group cross-tabulation						Chi-square value	p-value
			Group		Total		
			Group 1	Group 2			
Comorbidities	Present	Count (N)	11	13	24	0.225	0.635
		% Within comorbidities	45.80%	54.20%	100.0%		
	Absent	Count (N)	35	33	68		
		% Within comorbidities	51.50%	48.50%	100.0%		
Total		Count (N)	46	46	92		
		% Within comorbidities	50.00%	50.00%	100.0%		

Table 6 presents the outcomes related to burst abdomen (one week post-surgery), wound infection (one-week post-surgery), and incisional hernia (six weeks and six months post-surgery) for the two groups. Regarding burst abdomen, a total of three cases were reported, with two occurring in Group 1 and one in Group 2, translating to 66.67% of the cases in Group 1 and 33.33% in Group 2. The chi-square value is 0.022, and the p-value is 0.881, indicating no statistically significant difference in distribution.

**Table 6:** Relative incidence and analysis of complications in Group 1 and Group 2 Inferential statistical analysis using Pearson’s chi-square test p < 0.05 is statistically significant

Complications * Group 1 and 2 cross-tabulation						
Postoperative period	Complication		Group 1 (N = 46)	Group 2 (N = 46)	Chi-square value	p-value
One week	Burst abdomen	Yes	2 (66.67%)	1 (33.33%)	0.022	0.881
		No	44 (49.44%)	45 (50.56%)		
One week	Wound infection	Yes	10 (100%)	0 (0%)	11.22	0.001
		No	36 (43.9%)	46 (56.1%)		
Six weeks/six months	Incisional hernia	Yes	5 (83.33%)	1 (16.67%)	6.42	0.001
		No	41 (47.67%)	45 (50.56%)		

For wound infections, all 10 cases were found in Group 1, with none reported in Group 2. The chi-square value of 11.220 and a p-value of 0.001 indicate a significant difference between the two groups. In the case of incisional hernias, there were six cases total, with five occurring in Group 1 and one in Group 2, resulting in 83.33% of cases in Group 1 and 16.67% in Group 2. The chi-square value of 6.420 and a p-value of 0.001 further suggest a statistically significant difference between the two groups.

Table 7 illustrates the occurrence of localized fluid collection among patients undergoing different rectus closure techniques. In Group 1 (CRC), 32.6% of patients developed a localized collection, whereas only 10.9% of patients in Group 2 (IMSJ) experienced this complication. Notably, patients in both groups received the same type of dressing. This finding suggests that, compared to the traditional CRC method, the IMSJ technique is associated with a reduced incidence of localized fluid collections.

**Table 7:** Relative incidence of localized collection in Group 1 and Group 2 CRC, conventional rectus closure; IMSJ, interrupted modified Smead-Jones

Localized collection	Group 1 (CRC)	Group 2 (IMSJ)	Total
Yes	15 (32.6%)	5 (10.9%)	20
No	31 (67.4%)	41 (89.1%)	72
Total	46	46	92

**Figure 2:**
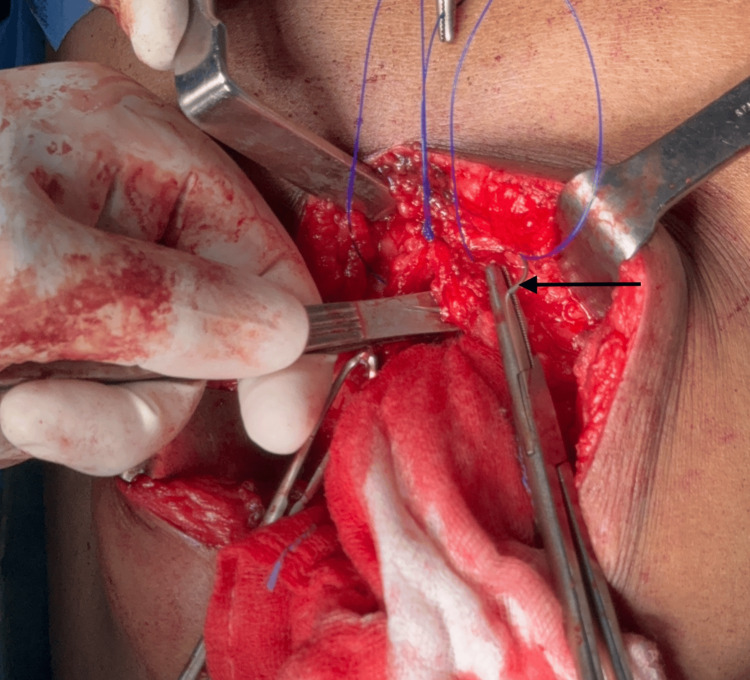
First suture, far bite taken from the same side rectus (pointer)

**Figure 3:**
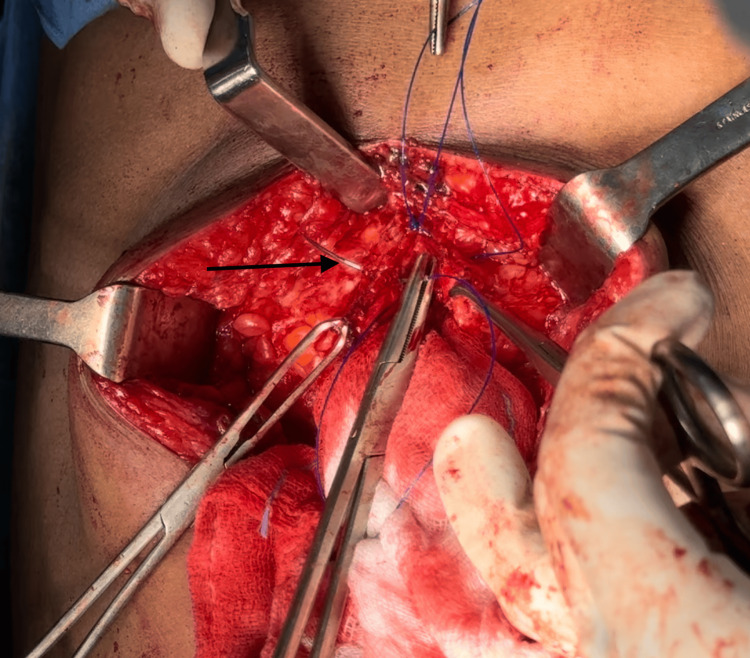
Second suture, near bite taken from the opposite side rectus (pointer)

**Figure 4:**
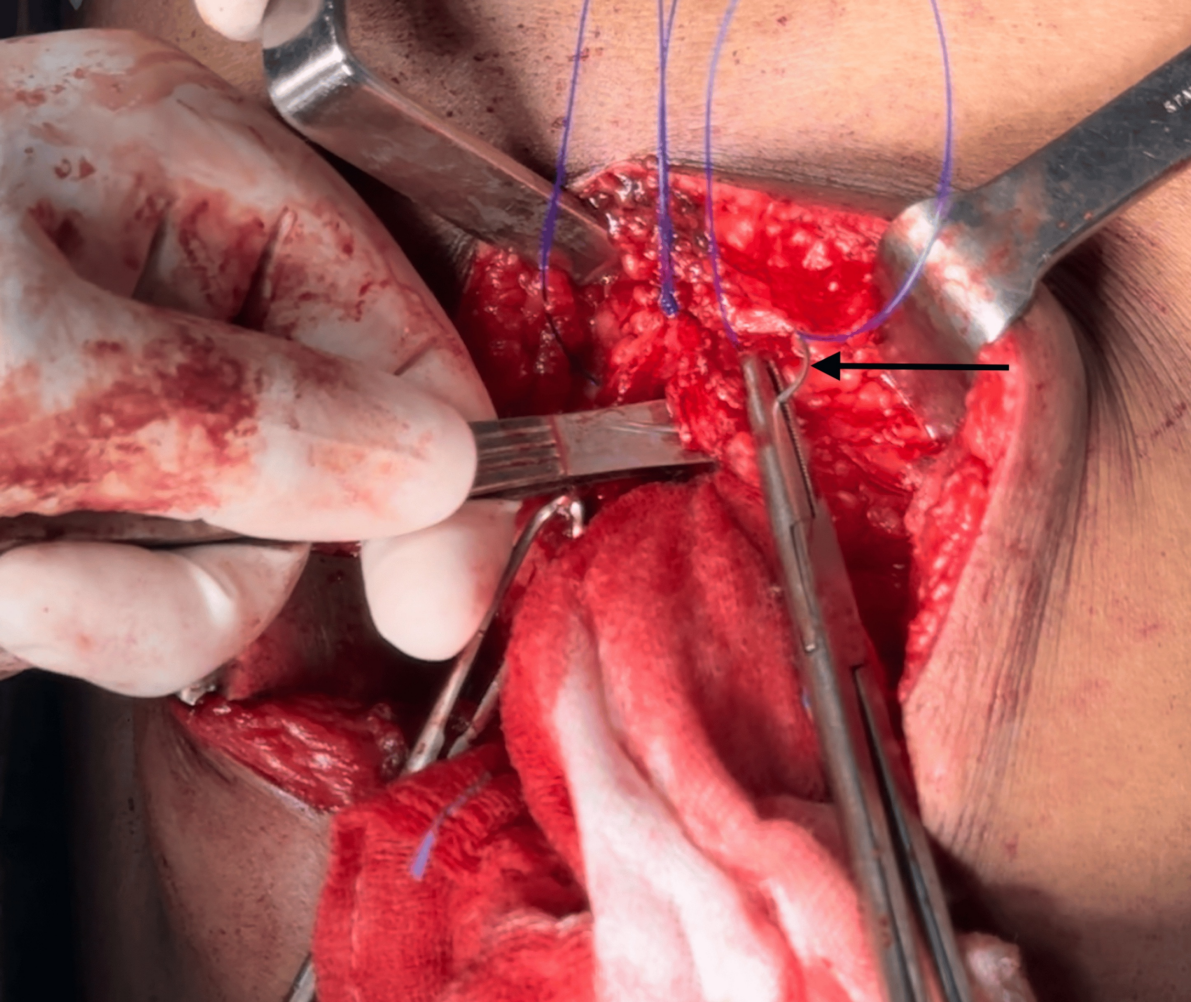
Third suture, near bite taken from the same side rectus (pointer)

**Figure 5:**
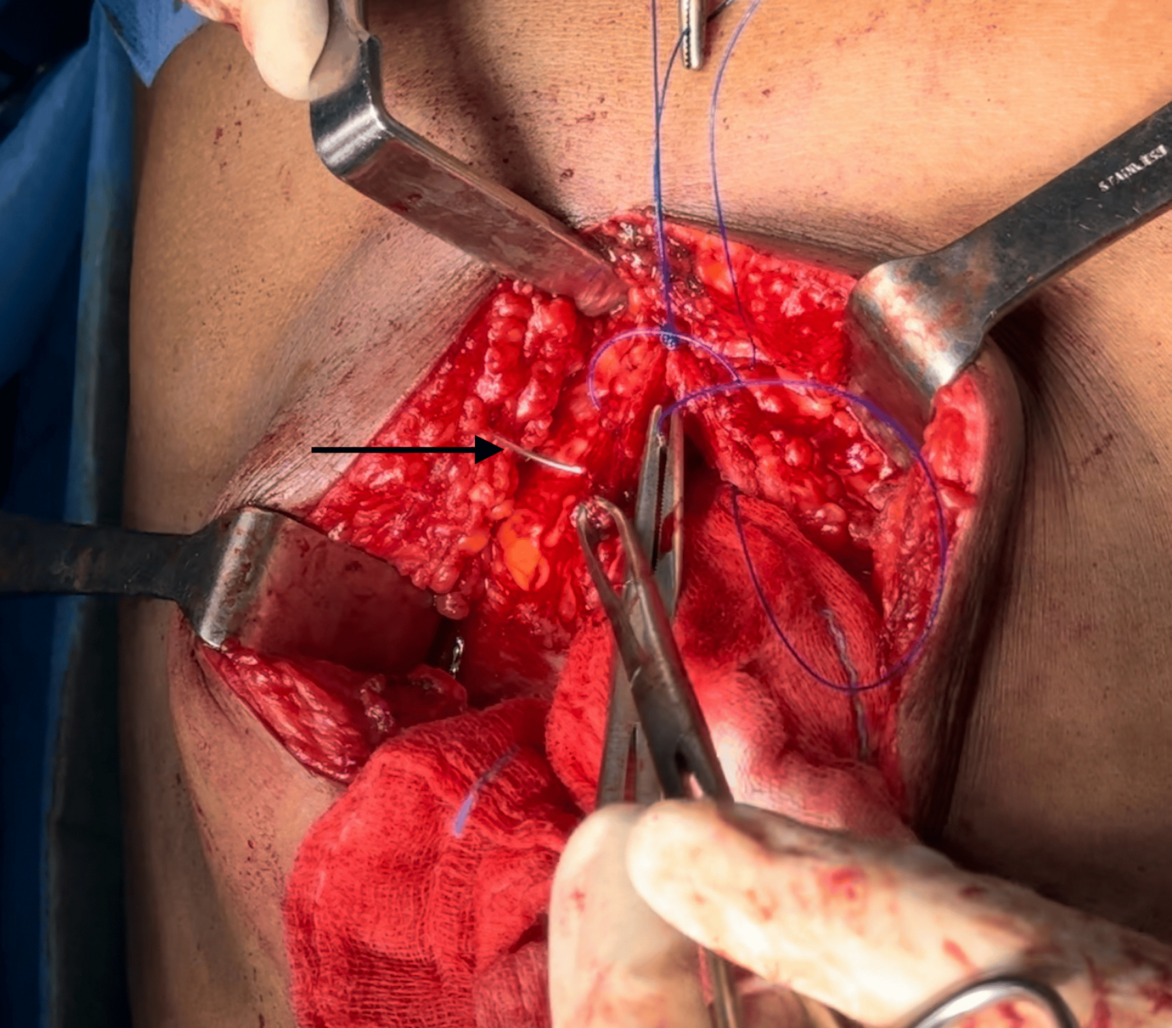
Fourth suture, far bite taken from the opposite side rectus (pointer)

The following figures provide representative images illustrating the IMSJ closure technique (Figure 2, Figure 3, Figure 4, Figure 5).

## Discussion

Duodenal perforation and RTAs with blunt abdominal injuries accounted for the highest number of cases in our study. Our main conclusions indicate no statistically significant difference in the distribution of burst abdomen between the two groups, with a total occurrence of three out of 96 patients. However, Group 1 had five occurrences (p = 0.001) of incisional hernia and 10 occurrences (p = 0.001) of wound infection, revealing a statistically significant difference between the groups regarding these complications.

Wound infections burst abdomens, and incisional hernias pose significant risks following laparotomy, affecting patient morbidity and mortality. Recent studies have noted an upward trend in wound dehiscence, particularly as more complex cases with multiple risk factors undergo emergency procedures for conditions like peritonitis. Such complications often arise from sudden increases in intra-abdominal pressure due to actions such as retching or prolonged coughing and straining during urination or defecation. Indicators of a potentially burst abdomen include serosanguinous discharge from the wound around the fourth to sixth postoperative day, visible bowel or omental tissue from the wound, and a sensation of abdominal heaviness or giving way.

Optimal fascial closure techniques are crucial for maintaining integrity throughout the healing process. The fascia of the anterior abdominal wall regains only one-fifth of its strength in the first two weeks. By the end of the first month, the fascia becomes self-supportive and regains half its normal tensile strength, with approximately eighty percent of its durability returning by four months post-surgery [4]. The risk of suture material tearing through the rectus fascia is inversely proportional to the suture’s cross-sectional diameter, with thicker sutures providing greater resistance. A meta-analysis of 23 randomized studies found that using interrupted sutures, compared to continuous suturing, reduced the incidence of burst abdomens by half [5].

The study group revealed a mean age of 53.45 ± 7.98 years, with the age range of 46-60 years comprising the largest proportion of patients. This age distribution aligns with previous findings in the literature, indicating that older patients, particularly those over 45 years, are more prone to complications following abdominal surgeries due to decreased tissue resilience and increased comorbidities [6,7]. In this study, the proportion of cases was higher in females (65.2%) compared to males (34.8%). This notable skew toward a higher female population may be influenced by the types of surgeries included in the study, as previous studies have suggested differences in surgical morbidity rates between genders [8,9].

However, the distribution of sexes between Group 1 and Group 2 showed no significant difference (chi-square value of 0.767 and a p-value of 0.381), indicating that sex does not significantly influence group allocation. This finding aligns with studies suggesting that while sex may influence specific surgical outcomes, it does not necessarily affect the choice of surgical technique [10,11].

In a study conducted by Agrawal et al. and similar studies comparing interrupted and conventional continuous closures in surgical and gynecological patients, the interrupted abdominal wall closure method was supported to prevent a burst abdomen. This study further recommended interrupted rectus closure as the preferred technique for all midline incisions, particularly for patients at increased risk of abdominal dehiscence, such as those with abdominal sepsis [12,13].

Interrupted suturing is favored over continuous suturing due to its reduced risk of bursting the abdomen, attributed to its “hacksaw” effect, which allows adjustment to stress and strain during physical movements. The Smead-Jones technique, involving sequential closure of individual fascial layers with interrupted knots, maintains fascial approximation even if one suture knot fails. In contrast, continuous rectus closure features a continuous suture line that can easily fail if any single suture loosens or breaks. This is a significant advantage of the Smead-Jones method over CRC [14].

The analysis of localized collection occurrences revealed a significantly higher incidence in the CRC group (32.6%) compared to the Smead-Jones group (10.9%). This notable difference in wound infection, incisional hernia, and localized collection underscores the effectiveness of the Smead-Jones technique.

The conventional method is prone to creating dead spaces where fluid can accumulate, leading to higher seroma rates. In contrast, the Smead-Jones technique, with its tension-distributing suture pattern and interrupted technique, allows fluid to drain away, minimizing retention and reducing the risk of seroma. This study highlights the Smead-Jones method as a superior choice for reducing postoperative incidences of incisional hernias, wound infections, and localized collection in midline laparotomy closures. However, the occurrence of burst abdomen was similar across both techniques.

Key limitations of this study include the small sample size and the lack of preoperative risk assessment. The study did not balance the number of patients undergoing emergency versus elective laparotomy, potentially skewing outcomes due to unaccounted confounding variables. Being an observational study with techniques chosen based on the surgeon’s discretion, it may lack the power to establish a direct cause-and-effect relationship, which could have been better addressed through a randomized controlled trial. Additionally, the skill required to learn and master the Smead-Jones technique can be considered a limitation. These factors necessitate careful consideration.

## Conclusions

This study contributes to the existing literature on midline laparotomy closure techniques, suggesting that the IMSJ method offers enhanced protection against early postoperative complications, such as wound infection and localized collection, as well as late complications like incisional hernias. Our findings indicate that the incidence of wound infection and incisional hernias is significantly lower with the IMSJ technique. This improvement is likely attributable to the technique’s superior tensile strength and more effective distribution of tension across the wound. Further research with larger sample sizes is needed to elucidate the nuances of these findings and potentially confirm the superior efficacy of the Smead-Jones method for specific patient demographics or clinical conditions.
